# Association of Circulating Apolipoprotein AI Levels in Patients With Alzheimer's Disease: A Systematic Review and Meta-Analysis

**DOI:** 10.3389/fnagi.2022.899175

**Published:** 2022-05-18

**Authors:** Jun-hui Tong, Shi-qiang Gong, Yan-song Zhang, Jian-ru Dong, Xin Zhong, Min-jie Wei, Ming-yan Liu

**Affiliations:** Department of Pharmacology, School of Pharmacy, China Medical University, Shenyang, China

**Keywords:** apolipoprotein AI, Alzheimer's disease, meta-analysis, serum, plasma

## Abstract

With the development of medicine, our research on Alzheimer's disease (AD) has been further deepened, but the mechanism of its occurrence and development has not been fully revealed, and there is currently no effective treatment method. Several studies have shown that apolipoprotein AI (ApoA-I) can affect the occurrence and development of Alzheimer's disease by binding to amyloid β (Aβ). However, the association between circulating levels of ApoA-I and AD remains controversial. We conducted a meta-analysis of 18 studies published between 1992 and 2017 to determine whether the ApoA-I levels in the blood and cerebrospinal fluid (CSF) are abnormal in AD. Literatures were searched in PubMed, EMBASE and Web of Science databases without language limitations. A pooled subject sample including 1,077 AD patients and 1,271 healthy controls (HCs) was available to assess circulating ApoA-I levels; 747 AD patients and 680 HCs were included for ApoA-I levels in serum; 246 AD patients and 456 HCs were included for ApoA-I levels in plasma; 201 AD patients and 447 HCs were included for ApoA-I levels in CSF. It was found that serum and plasma levels of ApoA-I were significantly reduced in AD patients compared with HCs {[standardized mean difference (SMD) = −1.16; 95% confidence interval (CI) (−1.72, −0.59); *P* = 0.000] and [SMD = −1.13; 95% CI (−2.05, −0.21); *P* = 0.016]}. Patients with AD showed a tendency toward higher CSF ApoA-I levels compared with HCs, although this difference was non-significant [SMD = 0.20; 95% CI (−0.16, 0.56); *P* = 0.273]. In addition, when we analyzed the ApoA-I levels of serum and plasma together, the circulating ApoA-I levels in AD patients was significantly lower [SMD = −1.15; 95% CI (−1.63, −0.66); *P* = 0.000]. These results indicate that ApoA-I deficiency may be a risk factor of AD, and ApoA-I has the potential to serve as a biomarker for AD and provide experimental evidence for diagnosis of AD.

**Systematic Review Registration:** PROSPERO, identifier: 325961.

## Introduction

Alzheimer's disease (AD) is a progressive and highly disabling neurodegenerative disease and there is currently no specific treatment. It is estimated that about 50 million people worldwide suffer from AD (Satizabal et al., 2016). In the coming decades, due to the extension of human life expectancy and the aging of the population, the prevalence will increase sharply (Prince et al., 2013), which makes the number of AD patients significantly increase.

At present, a clinical diagnosis of AD is only considered possible, besides, the error rate of diagnosis based solely on clinical standards is nearly 40% (Holmes, 2014). Only by conducting histopathological examination of the brain at autopsy can we make a definite diagnosis of AD (Dubois et al., 2007). Due to the difficulty of diagnosis, AD patients can only be diagnosed with dementia when they are seriously ill. With the rapid development of biomarkers (Blennow and Zetterberg, 2018), especially lipid biomarkers (Wong et al., 2017), our thinking on the diagnosis and prevention of AD is also changing.

Apolipoproteins are the physiological agents for the transport along the body of aqueous fluids of the hydrophobic lipids (Fuior and Gafencu, 2019). Apolipoprotein E (ApoE), a member of the apolipoprotein family, has a well-established role in Aβ metabolism. The circulating ApoE level has also been found to be a potential biomarker of AD (Talwar et al., 2014). As a different subtype of the same family, apolipoprotein AI (ApoA-I) is the main apolipoprotein of high-density lipoprotein, which is involved in the reverse transport of cholesterol. This function may be affected by ApoA-I gene polymorphism, causing changes in brain cholesterol metabolism, thereby influencing the risk of early-onset non-familiar AD (Vollbach et al., 2005). Studies have shown that the A allele of ApoA1-75bp G/A polymorphism increases the risk of AD in people aged 66 and younger, and promotes earlier onset of the disease (Vollbach et al., 2005). To date, several studies have evaluated the levels of ApoA-I in the serum, plasma, and cerebrospinal fluid (CSF) of AD patients and provided evidence of their correlation with AD risk. These results greatly increase the possibility of ApoA-I as a potential biomarker of AD.

However, current studies evaluating ApoA-I levels have yielded conflicting results about the relationship between ApoA-I levels and AD risk. Some studies have shown that circulating ApoA-I levels in AD patients are decreased (Merched et al., 2000), while other studies have shown that circulating ApoA-I levels in AD patients are increased (Kuriyama et al., 1992,Demeester et al., 2000). And most studies only study the changes of ApoA-I at a single level in serum, plasma or cerebrospinal fluid, and do not take into account the blood and cerebrospinal fluid. Therefore, we conducted a comprehensive meta-analysis to compare the levels of ApoA-I in serum, plasma, and cerebrospinal fluid between AD patients and healthy controls (HCs) to clarify whether there is a significant relationship. To our knowledge, this is the first meta-analysis of studies on the association of circulating ApoA-I levels with AD.

## Materials and Methods

### Literature Search

The review was conducted in accordance with the “Preferred Reporting Items for Systematic reviews and Meta-Analyses” (PRISMA) statement (Moher et al., 2009). A comprehensive search strategy was applied using PubMed, EMBASE and Web of Science databases. Keywords including “Apolipoprotein A-I,” “ApoA-I,” “Alzheimer's disease,” “AD,” and “Dementia” were searched for related studies that assessed ApoA-I levels in AD patients and HCs, which included all published studies dating from the inception of databases to December 8, 2021.

### Inclusion and Exclusion Criteria

Inclusion criteria. (1) human subjects; (2) case-control study design; (3) both AD and control groups described in terms of sample size and ApoA-I concentration in serum, blood, plasma, or CSF; (4) The reported ApoA-I level is the average of the standard deviation (SD), standard error (SE) or the median; (5) For studies with stratified groups of AD patients, we treated each stratum as a separate study.

Exclusion criteria. (1) letter, review, or case reports; (2) Incomplete or unavailable data; (3) The subjects do not meet the inclusion criteria; (4) studies lacking quantitative data on ApoA-I concentrations.

### Literature Screening and Data Extraction

The following process was carried out by two researchers alone. Unqualified literatures were initially excluded by screening title and abstract, and the included documents were determined by reading the full text. Cross-check the final included research results. If there is no consensus on the existing differences, a third researcher needs to be addressed to solve the problem, and the final reasonable data was determined by the corresponding author. The nine-star Newcastle-Ottawa Scale (NOS) was used for quality assessment.

### Statistical Analysis

Statistical analyses were performed using Stata 12.0 software for meta-analysis. For continuous variable data, standardized mean difference (SMD) and 95% confidence interval (CI) were calculated as the statistics for effect analysis. The test of statistical heterogeneity applies the χ^2^ test. If there is no statistical heterogeneity (*P* > 0.10, *I*^2^ ≤ 50%), the fixed effects model is used; otherwise, the random effects model is adopted for analysis.

Subgroup analyses were then used to assess possible sources of heterogeneity, estimating the effect of differences in subject populations and methods of ApoA-I measurement. Egger's and Begg's tests were conducted to assess whether there is publication bias and sensitivity analysis was performed to explore whether single difference could markedly influence the overall outcome.

## Results

### Selection of Studies

**Figure 1:**
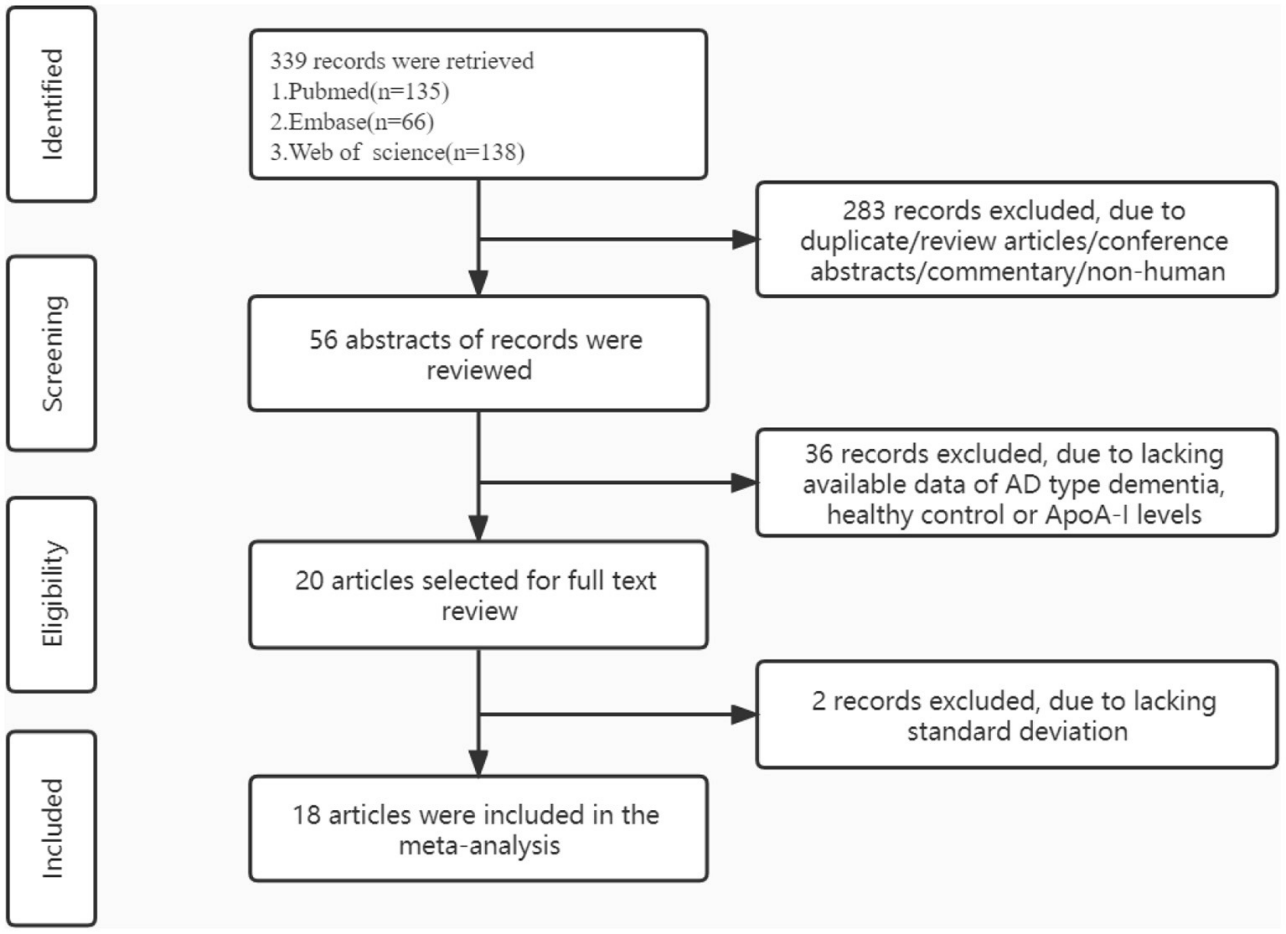
Flow diagram of study selection.

After the literature search, a total of 339 related articles were obtained, including 135 in Pubmed, 66 in EMBASE and 138 in Web of Science. After reading the title and abstract and removing duplicates, 56 papers were initially included. After eliminating the literature by reading the full text, 36 records were excluded (due to lacking available data of AD type dementia, healthy control or ApoA-I levels) and 18 literatures were finally identified, the literature retrieval flow chart is shown in Figure 1.

In the included studies, the sample sizes varied between 25 and 429. The subjects were between 58.5 ± 4.0 and 83.77 ± 1.19 years old, with the proportion of female subjects between 23 and 87%. Mean age was omitted from three studies. In addition, five studies lacked diagnostic criteria for AD. Of all included studies, 11 were from Asia, three were from Europe, and four were from North America. Regarding the measurement method used to measure ApoA-I levels, eight studies used the ELISA assays, five studies used Immunonephelometry assays, one study used MALDI-TOF MS assays and four did not reported (NR) specify assays. Some studies showed additional grouping information of AD patients, including CDR score, low and high Aβ, ApoE3/3 and ApoE4/3, and we considered each of them as an independent study in the meta-analysis. The basic information of the literature that met the inclusion requirements is shown in Table 1. The NOS quality assessment scale is shown in Supplementary Table 1. Most included studies were of high quality (15 high-quality and three moderate-quality studies).

**Table 1:** Baseline characteristics of the included trials.

**References**	**Year**	**Population**	**AD diagnostic criteria**	**Study design**	**MMSE score in AD**	**Assay method**	**Diagnosis (*n*)**		**Mean age (y)**	**Male/female**	**ApoA-I levels (mg/dl)**	***P*-value**	**Sample source**
Kuriyama et al. (1992)	1992	Japan (A)	DSM-III-R	Control		ELISA	HCs	21	63.3 ± 8.6	NR	106 ± 30	NS	Serum
				Case-	NR		AD	22	72.9 ± 4.6	NR	118 ± 26		
Kuriyama et al. (1994)	1994	Japan (A)	NINCDS-ADRDA	Control		Immunoturbidity method	HCs	45	69.6 ± 8.9	18/27	138.7 ± 41.6	<0.001	Serum
				Case-	NR		AD	44	75.4 ± 6.0	7/37	114.8 ± 23.1		
Merched et al. (2000)	2000	France(C)	NINCDS-ADRDA	Control		Immunonephelometry	HCs	59	75.37 ± 5.27	28/31	1.65 ± 0.26	<10 ^−7^	Serum
			DSM-III-R	Case-	NR		AD	98	77.56 ± 8.85	28/70	1.34 ± 0.31		
							HCs ApoE3/3	38			1.61 ± 0.25	NR	
							AD ApoE3/3	40			1.28 ± 0.29		
							HCs ApoE4/3	12			1.65 ± 0.26	NR	
							AD ApoE4/3	39			1.38 ± 0.34		
Yamamoto et al. (2005)	2005	Japan (A)	NINCDS-ADRDA	Control		Turbidimetric immunoassays	HCs	32	77 ± 5	17/15	137 ± 26	NS	Serum
				Case-	11 ± 7		AD	61	80 ± 6	24/37	130 ± 23		
Liu et al. (2006)	2006	China (A)	NR	Control		MALDI-TOF MS	HCs	74	71.5 ± 4.5	26/48	144.53 ± 19.91	<0.0002	Serum
				Case-	NR		AD	59	72.3 ± 7.2	20/39	112.29 ± 21.33		
Xiao et al. (2012)	2012	China (A)	NINCDS-ADRDA	Control		Immunoturbidimetric	HCs	104	76.5 ± 6.14	56/48	1.21 ± 0.22	NS	Serum
				Case-	NR		AD	104	77.8 ± 6.74	57/47	1.17 ± 0.28		
Lin et al. (2015)	2015	China (A)	NINCDS-ADRDA	Control		ELISA	HCs CDR 0	160	NR	NR	0.140 ± 0.040	<0.05	Serum
				Case-	19.8 ± 4.9		AD CDR 0.5	84	NR	NR	0.078 ± 0.016		
							HCs CDR 0	160	NR	NR	0.140 ± 0.040	<0.05	
							AD CDR 1	36	NR	NR	0.055 ± 0.015		
							HCs CDR 0	160	NR	NR	0.140 ± 0.040	<0.05	
							AD CDR 2	27	NR	NR	0.035 ± 0.015		
Choi et al. (2016)	2016	Korea (A)	NR	Control	27.2 ± 2.4	Immunoturbidimetry	HCs	35	71.4 ± 5.3	12/23	136.2 ± 27.5	<0.001	Serum
				Case-	23.5 ± 2.7		AD (low Aβ)	13	69.5 ± 5.9	2/11	109.4 ± 15.7		
				Control	27.2 ± 2.4		HCs	35	71.4 ± 5.3	12/23	136.2 ± 27.5	<0.001	
				Case-	23.1 ± 3.7		AD (high Aβ)	15	71.9 ± 8.4	2/13	130.7 ± 15.2		
Ya and Lu (2017)	2017	China (A)	NINCDS-ADRDA	Control		ELISA	HCs	100	64.85 ± 5.88	50/50	1.52 ± 0.13	<0.05	Serum
			DSM-IV-R	Case-	17.38 ± 5.53		AD	105	69.94 ± 4.45	60/45	1.04 ± 0.15		
Kawano et al. (1995)	1995	Japan (A)	NR	Control		NR	HCs ApoE3/3	67	76.0 ± 5.5	26/41	148.3 ± 28.2	<0.0001	Plasma
				Case-	NR		AD ApoE3/3	16	77.5 ± 4.3	5/11	117.4 ± 15.3		
							HCs ApoE4/3	12	71.8 ± 14.8	5/7	143.4 ± 28.3	<0.02	
							AD ApoE4/3	29	74.7 ± 5.6	11/18	119.8 ± 22.6		
Bergt et al. (2006)	2006	USA (C)	NINCDS-ADRDA	Control		ELISA	HCs	20	75 ± 7	10/10	268 ± 70	NS	Plasma
				Case-	NR		AD	20	78 ± 10	10/10	263 ± 70		
Khalil et al. (2012)	2012	Canada (C)	NINCDS-ADRDA	Control		NR	HCs	20	74.71 ± 1.40	7/13	1.69 ± 0.07	<0.001	Plasma
			DSM-IV	Case-	Mild: 26.20 ± 1.97 (*n* = 14)		AD	39	83.77 ± 1.19	5/34	1.35 ± 0.08		
					Moderate: 19.14 ± 3.25 (*n* = 14)								
					Severe: 11.27 ± 2.45 (*n* = 11)								
Yang et al. (2015)	2015	China (A)	NINDS-AIREN	Control		NR	HCs	25	72.00 ± 6.69	7/18	1.58 ± 0.45	NR	Plasma
				Case-	21.33 ± 2.24		AD	25	71.92 ± 7.28	10/15	1.38 ± 0.20		
Slot et al. (2017)	2017	Netherland (C)	NIA-AA	Control		ELISA	HCs	312	62.4 ± 8.8	190/122	1.4 ± 0.3	NS	Plasma
				Case-	26.5 ± 2.5		AD	117	68.7 ± 7.9	59/58	1.4 ± 0.3		
Song et al. (1997)	1997	Japan (A)	NINCDS-ADRDA	Control		ELISA	HCs	23	61.7 ± 10.3	10/13	0.0037 ± 0.0018	NS	CSF
			DSM-III-R	Case-	NR		EOAD	11	58.5 ± 4.0	5/6	0.0028 ± 0.0030		
							HCs	23	61.7 ± 10.3	10/13	0.0037 ± 0.0018	NS	
							LOAD	15	75.8 ± 4.4	4/11	0.0035 ± 0.0021		
Kindy et al. (1999)	1999	USA (C)	NR	Control		NR	HCs	10	NR	NR	1.4 ± 0.7	NR	CSF
				Case-	NR		AD	15	NR	NR	1.6 ± 0.8		
Demeester et al. (2000)	2000	Belgium(C)	NINCDS-ADRDA	Control		ELISA	HCs	55	NR	NR	0.96 ± 0.47	NR	CSF
			DSM-IV	Case-	NR		AD	17	NR	NR	1.53 ± 0.60		
Yassine et al. (2016)	2016	USA (C)	NR	Control		ELISA	HCs	47	78 ± 7	20/27	1.76 ± 1.3	NS	CSF
				Case-	15 ± 8		AD	26	77 ± 10	9/17	1.74 ± 1.1		
Slot et al. (2017)	2017	Netherland (C)	NIA-AA	Control		ELISA	HCs	312	62.4 ± 8.8	190/122	0.0034 ± 0.0017	NS	CSF
				Case-	26.5 ± 2.5		AD	117	68.7 ± 7.9	59/58	0.0038 ± 0.0021		

*AD, Alzheimer's disease; HCs, healthy controls; ApoA-I, Apolipoprotein A-I; A, Asian, C, Caucasian; ELISA, enzyme-linked immunosorbent assay; MALDI-TOF MS, Matrix-Assisted Laser Desorption/Ionization Time-of-Flight Mass Spectrometry; NINCDS–ADRDA, National Institute of Neurological and Communicative Disorders and Stroke–Alzheimer's Disease and Related Disorders Association; DSM III, Diagnostic and Statistical Manual of Mental Disorders, 3rd Edition; DSM-IV, Diagnostic and Statistical Manual of Mental Disorders, 4th Edition; CSF, Cerebrospinal fluid; CDR, Clinical Dementia Rating; low Aβ, aMCI-; high Aβ, aMCI+; aMCI, amnestic mild cognitive impairment, Based on Pittsburgh Compound B (PiB) retention at baseline, the aMCI subjects were divided into low Aβ (aMCI-) and high Aβ (aMCI+) subgroups; MMSE, Mini Mental State Examination; LOAD, Late onset AD; EOAD, Early onset AD; NS, not significant; NR, Not reported*.

### Meta-Analysis of Serum ApoA-I Levels Between AD Patients and HCs

**Figure 2:**
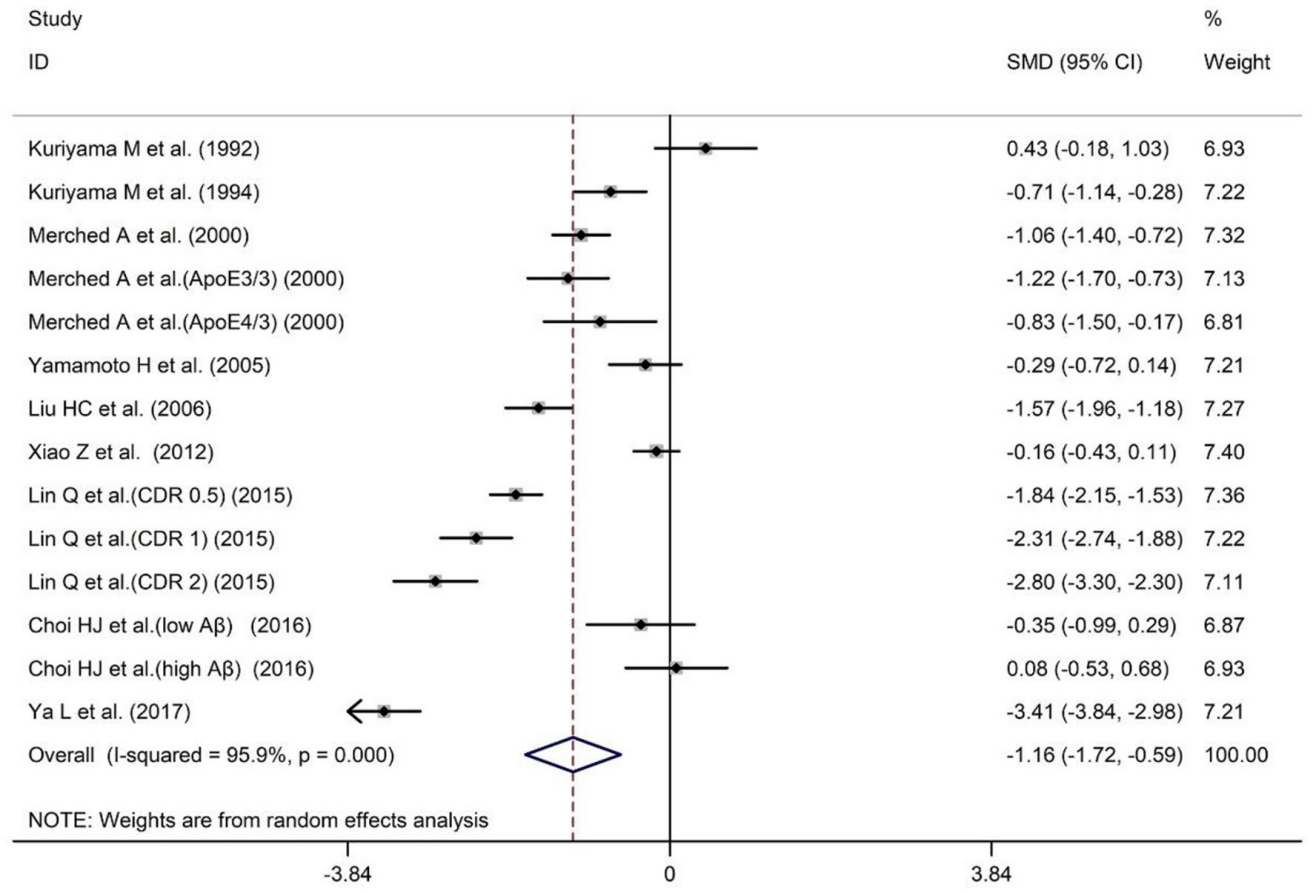
Forest plot of random-effects meta-analysis of differences in serum ApoA-I between AD and HCs. SMD, standardized mean difference; CI, confidence interval.

A total of nine studies have reported different serum ApoA-I levels in AD patients and HCs. The sample size contained 747 AD patients and 680 HCs (Table 1). The results showed that AD patients had significantly lower serum ApoA-I compared with HCs [SMD = −1.16; 95% CI (−1.72, −0.59); *P* = 0.000; Figure 2]. In addition, there was high heterogeneity among the nine studies [*I*^2^ = 95.9%, *P* = 0.000]. To explore the source of heterogeneity, we conducted subgroup analyses based on differences in subject population and the methods of ApoA-I measurement (Table 2). And in population subgroup analysis, ApoA-I levels in serum of Asian AD patients were significantly lower than HCs [SMD = −1.18; 95% CI (−1.90, −0.47); *P* = 0.001; Table 2]. Serum ApoA-I levels in Caucasian AD patients were also significantly lower than in HCs [SMD = −1.07; 95% CI (−1.33, −0.81); *P* = 0.000; Table 2]. In addition, there is still high heterogeneity among studies in Asian population (*I*^2^ = 96.8%, *P* = 0.000), while there is no heterogeneity among studies in Caucasian population (*I*^2^ = 0.0%, *P* = 0.659), indicating that subject populations may be a source of heterogeneity (Table 2). Subgroup analysis according to the methods of ApoA-I measurement also showed significant heterogeneity (Table 2), suggesting that the methods of ApoA-I measurement was not a major source of heterogeneity. The results of sensitivity analysis showed that the results of all outcome indicators were relatively stable, suggesting that the results were stable (Supplementary Table 2). Begg's (*P* = 0.743) and Egger's (*P* = 0.955) tests indicated no publication bias (Supplementary Tables 3A,B), indicating that the results are statistically robust.

**Table 2:** The subgroup analysis of studies reporting serum ApoA-I levels.

**Subgroups**	***n* of studies**	**SMD 95% (Cl)**	***I* ^2^**	***P*-value**
All studies	9	−1.16 (−1.72, −0.59)	95.9%	0.000
**Populations**
Asian	8	−1.18 (−1.90, −0.47)	96.8%	0.000
Caucasian	1	−1.07 (−1.33, −0.81)	0.0%	0.659
**Methods**
ELISA	3	−2.00 (−3.04, −0.96)	96.5%	0.000
Immunonephelometry	5	−0.57 (−0.91, −0.24)	77.3%	0.000
MALDI-TOF MS	1	−1.57 (−1.96, −1.18)	–	–

### Meta-Analysis of Plasma ApoA-I Levels Between AD Patients and HCs

**Figure 3:**
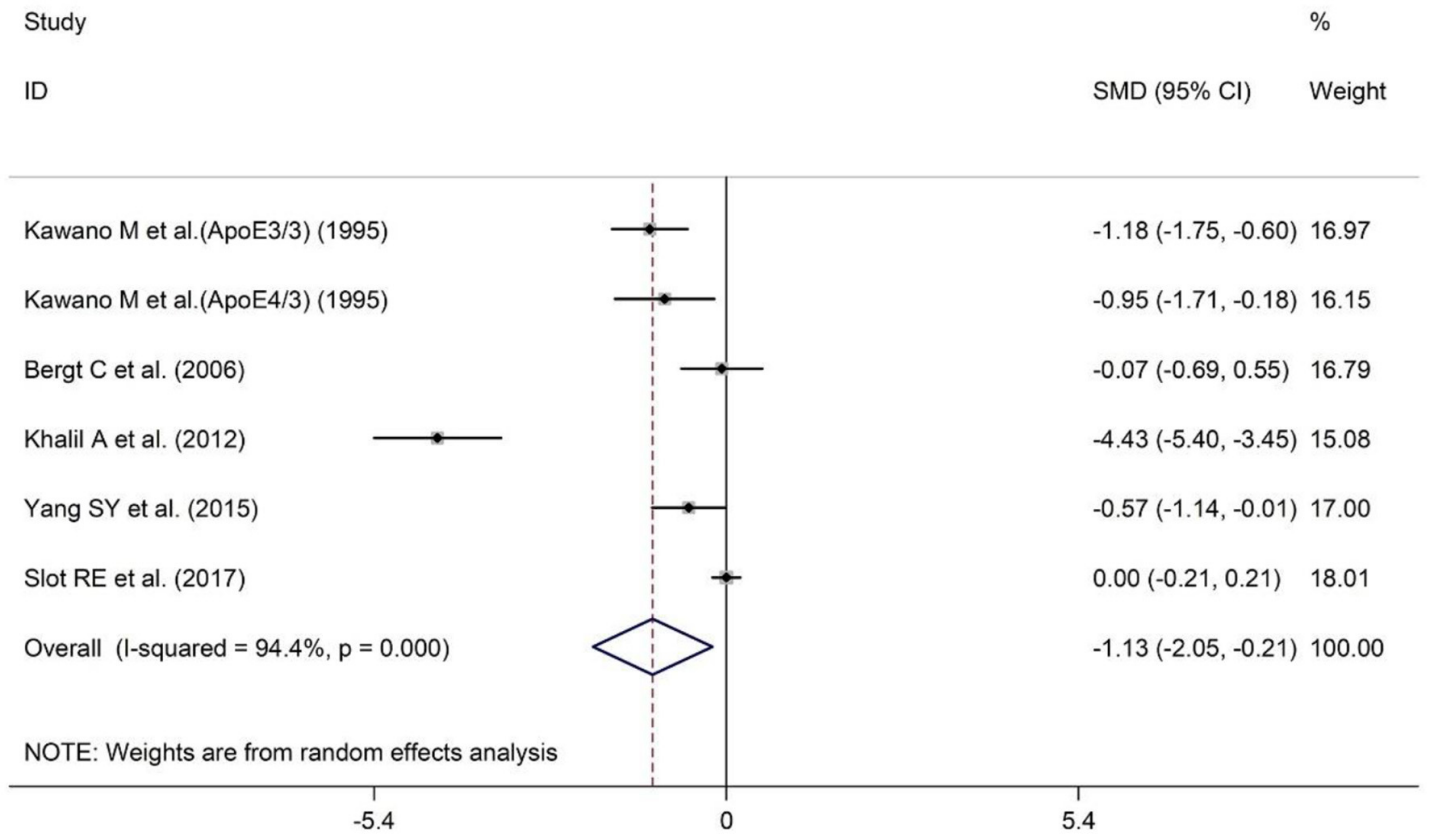
Forest plot of random-effects meta-analysis of differences in plasma ApoA-I between AD and HCs. SMD, standardized mean difference; CI, confidence interval.

A total of five studies have reported different plasma ApoA-I levels in AD patients and HCs. The sample size contained included 246 AD patients and 456 HCs (Table 1). The results indicated that AD patients had less ApoA-I levels in plasma than HCs [SMD = −1.13; 95% CI (−2.05, −0.21); *P* = 0.016; Figure 3]. Heterogeneity among the included studies was observed (*I*^2^ = 94.4%, *P* = 0.000). In the population subgroup analysis, the plasma ApoA-I level of AD patients in Asian population was significantly lower than that of HCs [SMD = −0.89; 95% CI (−1.26, −0.52); *P* = 0.000; Table 3]. Plasma ApoA-I levels also tended to decrease in AD patients in the Caucasian population compared with HCs, but the difference was not significant [SMD = −1.44; 95% CI (−3.42, −0.55); *P* = 0.155; Table 3]. In addition, there was no heterogeneity among the studies in the Asian population (*I*^2^ = 7.5%, *P* = 0.339), while there was still a high degree of heterogeneity among the studies in the Caucasian population (*I*^2^ = 97.4%, *P* = 0.000), indicating that subject populations may be a source of heterogeneity (Table 3). Subgroup analysis according to the method of ApoA-I measurement showed that ApoA-I levels in the plasma of AD patients in the NR group were significantly lower than those in HCs [SMD = −1.73; 95% CI (−3.10, −0.37); *P* = 0.013; Table 3]; There was no significant difference in plasma ApoA-I levels between AD patients in the ELISA group compared with HCs [SMD = −0.01; 95% CI (−0.21, 0.19); *P* = 0.942; Table 3]. In addition, there was still a high degree of heterogeneity among the studies in the NR group (*I*^2^ = 93.6%, *P* = 0.000), whereas there was no heterogeneity among the studies in the ELISA group (*I*^2^ = 0.0%, *P* = 0.831), which suggests that the method of ApoA-I measurement may also be a source of heterogeneity (Table 3). The results of sensitivity analysis showed that the results of all outcome indicators were relatively stable, suggesting that the results were stable (Supplementary Table 4). Begg's (*P* = 0.133) and Egger's (*P* = 0.075) tests indicated no publication bias (Supplementary Tables 5A,B), indicating that the results are statistically robust.

**Table 3:** The subgroup analysis of studies reporting plasma ApoA-I levels.

**Subgroups**	***n* of studies**	**SMD (95% Cl)**	***I* ^2^**	***P*-value**
All studies	5	−1.13 (−2.05, −0.21)	94.4%	0.000
**Populations**
Asian	2	−0.89 (−1.26, −0.52)	7.5%	0.339
Caucasian	3	−1.44 (−3.42, 0.55)	97.4%	0.000
**Methods**
NR	3	−1.73 (−3.10, −0.37)	93.6%	0.000
ELISA	2	−0.01 (−0.21, 0.19)	0.0%	0.831

### Meta-Analysis of Peripheral Blood ApoA-I Levels Between AD and HCs

**Figure 4:**
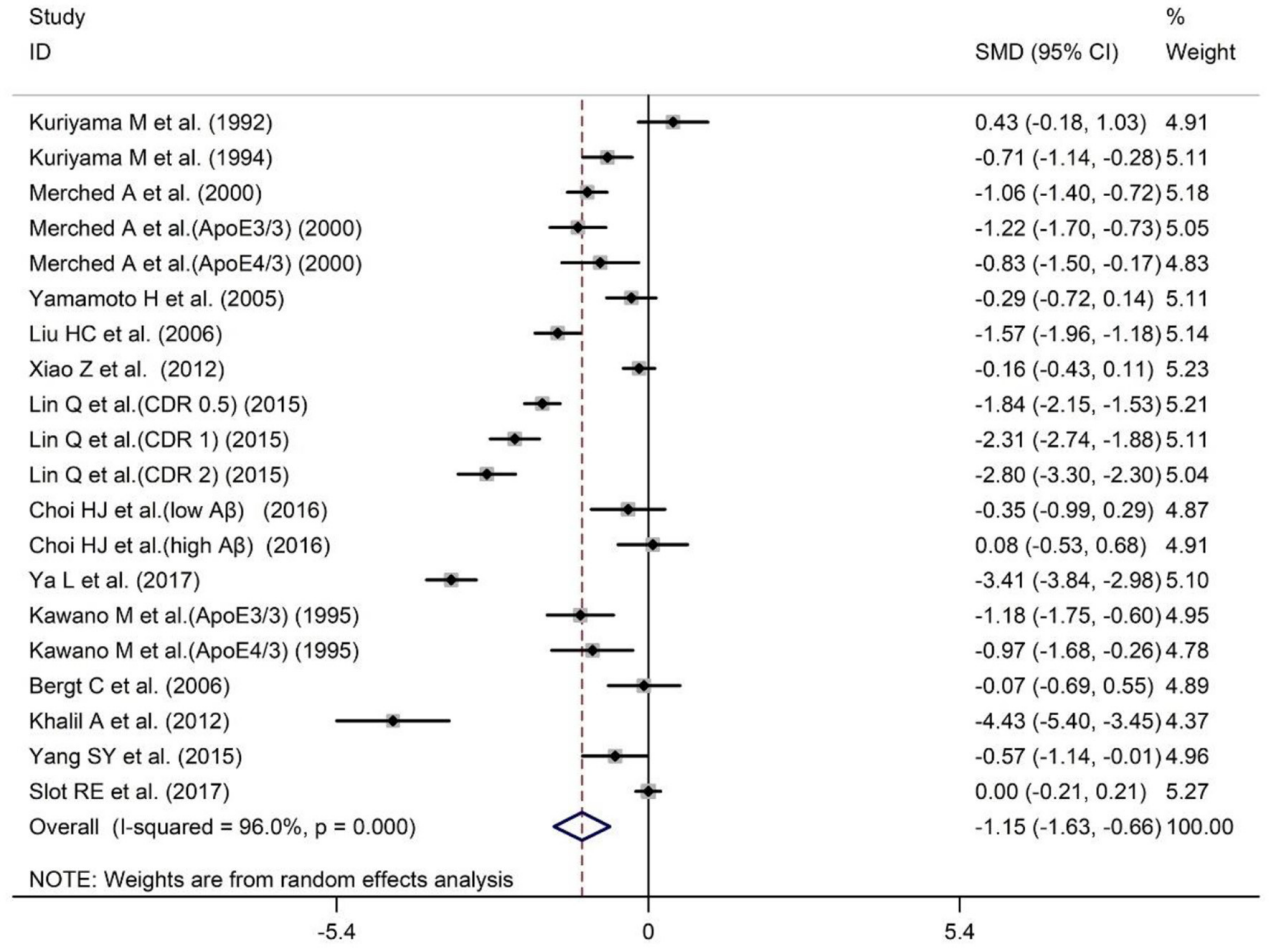
Forest plot of random-effects meta-analysis of differences in peripheral blood ApoA-I between AD and HCs. SMD, standardized mean difference; CI, confidence interval.

Serum and plasma are the most commonly used sources of peripheral blood samples. In order to verify whether the results of the overall analysis of peripheral blood ApoA-I levels are consistent with the analysis of serum or plasma alone, we combined the data of serum and plasma for analysis. The sample size of the 14 studies included 2,129 subjects, including 993 AD patients and 1,136 HCs (Table 1). The results revealed that peripheral blood ApoA-I levels in AD patients were significantly decreased compared with that in HCs [SMD = −1.15; 95% CI (−1.63, −0.66); *P* = 0.000; Figure 4], in addition to high heterogeneity among these studies (*I*^2^ = 96.0%, *P* = 0.000). Analysis of subgroups according to subject population and ApoA-I measurement method also showed significant heterogeneity (Table 4), suggesting that neither subject population nor ApoA-I measurement methods were the primary sources of heterogeneity. The results of sensitivity analysis showed that the results of all outcome indicators were relatively stable, suggesting that the results were stable (Supplementary Table 6). Begg's (*P* = 0.820) and Egger's (*P* = 0.187) tests indicated no publication bias (Supplementary Tables 7A,B), indicating that the results are statistically robust.

**Table 4:** The subgroup analysis of studies reporting peripheral blood ApoA-I levels.

**Subgroups**	***n* of studies**	**SMD (95% Cl)**	***I* ^2^**	***P*-value**
All studies	14	−1.15 (−1.63, −0.66)	96.0%	0.000
**Populations**				
Asian	10	−1.13 (−1.72, −0.53)	96.0%	0.000
Caucasian	4	−1.19 (−2.02, −0.35)	95.2%	0.000
**Methods**				
ELISA	5	−1.43 (−2.55, −0.32)	98.3%	0.000
Immunonephelometry	5	−0.57 (−0.91, −0.24)	77.3%	0.000
MALDI-TOF MS	1	−1.57 (−1.96, −1.18)	–	–
NR	3	−1.74 (−3.07, −0.40)	93.6%	0.000

### Meta-Analysis of CSF ApoA-I Levels Between AD and HCs

**Figure 5:**
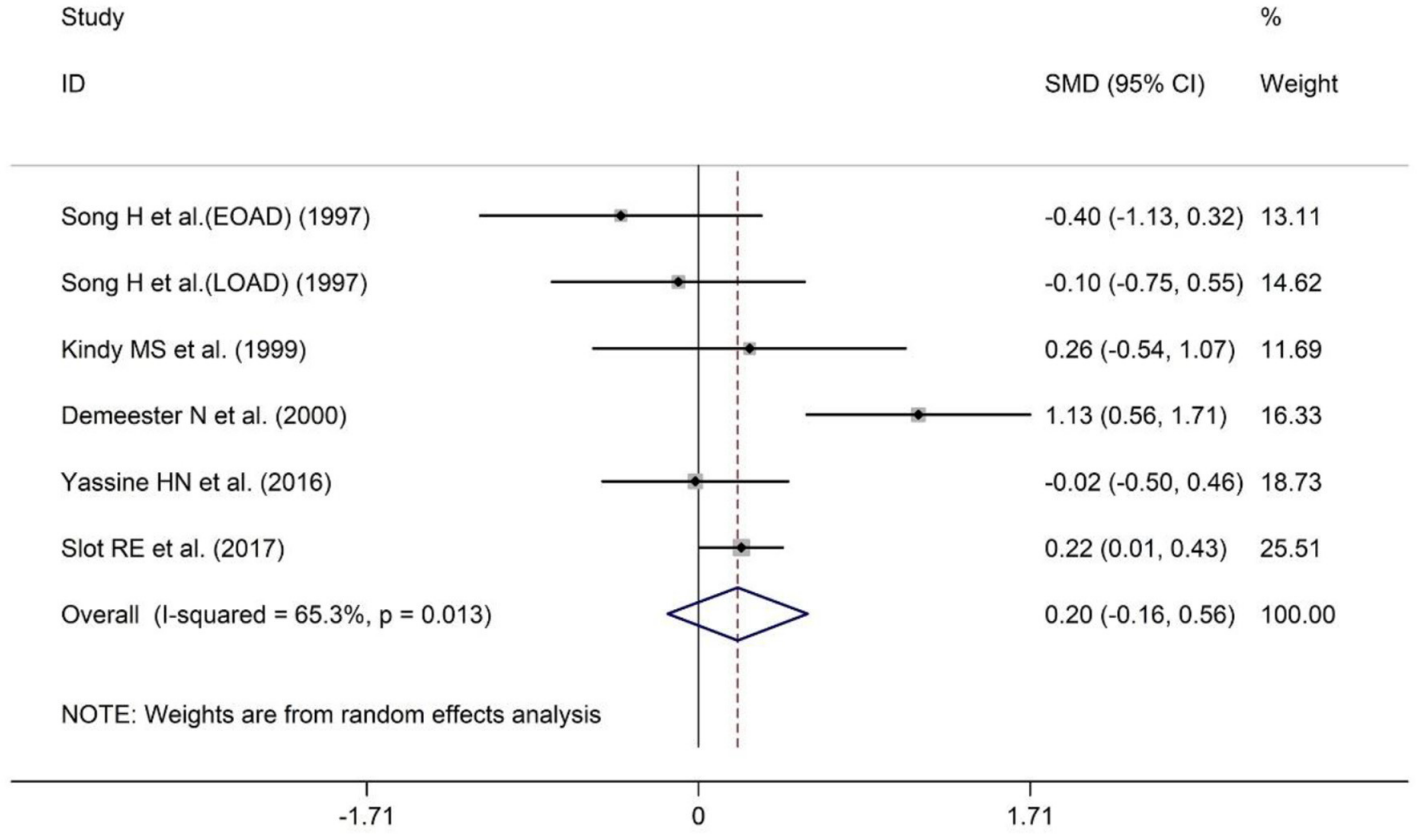
Forest plot of random-effects meta-analysis of differences in CSF ApoA-I between AD and HCs. SMD, standardized mean difference; CI, confidence interval.

A total of five studies have reported comparisons of CSF ApoA-I levels between AD patients and HCs. The sample size included 201 AD patients and 447 HCs (Table 1). Patients with AD showed a tendency toward higher CSF ApoA-I levels compared with HCs, although this difference was non-significant [SMD = 0.20; 95% CI (−0.16, 0.56); *P* = 0.273; Figure 5], so further analysis was not conducted.

## Discussion

Studies have shown that the deposition of Aβ in the human brain is slow and long-lasting, possibly for more than two decades (Villemagne et al., 2013). In other words, individuals with normal cognition may already have abnormal Aβ, which is the conceptual basis of preclinical AD (Vos and Visser, 2018), and opens up new possibilities for studying the development of AD and ultimately preventing dementia. Therefore, if we can identify circulating biomarkers related to the occurrence and development of AD, it will be more helpful to the clinical diagnosis and treatment of AD.

ApoA-I, the main protein component of plasma high-density lipoprotein (HDL), is not synthesized in the central nervous system (CNS) (Demeester et al., 2000; Koch et al., 2001). ApoA-I traverses the blood-brain barrier through endocytosis mediated by clathrin and cholesterol, suggesting that Blood-Brain Barrier (BBB) is an essential gateway for ApoA-I delivery to the brain (Zhou et al., 2019). Low high-density lipoprotein cholesterol (HDL-C) levels have been proven to be associated with cognitive impairment and various neurodegenerative diseases (Vitali et al., 2014). Study has shown that high plasma levels of HDL and ApoA-I can reduce the risk of AD (Robert et al., 2017). In addition, protein lipidation is a unique co-translational or posttranslational modification in which lipid moieties are covalently attached to proteins. And protein lipidation plays a critical role in cell signaling, and dynamically regulates protein functions in response to extrinsic and intrinsic cues (Chen et al., 2018). The extent of human ApoA-I lipidation strongly affects the Aβ efflux across the blood-brain barrier *in vitro* and ApoA-I in a disc-like conformation in the lipidation state may clear Aβ from the brain, thereby playing a protective role in the AD pathogenesis (Dal Magro et al., 2019). The interaction of ApoA-I with ATP-binding cassette A1 (ABCA1) could activate signaling molecules that modulate posttranslational ABCA1 activity or lipid transport activity (Zhao et al., 2012). The key signaling molecules in these processes include protein kinase A (PKA) (Jeon et al., 2010), protein kinase C (PKC) (Yamauchi et al., 2004), Janus kinase 2 (JAK2) (Tang et al., 2004, 2006), Rho GTPases (Hirano et al., 2000; Tsukamoto et al., 2001; Okuhira et al., 2010) and Ca^2+^ (Takahashi and Smith, 1999).

It is reported that binding of ApoA-I to Aβ can decrease Aβ aggregation, so it can affect the morphology of amyloid aggregates and protect neurons from Aβ-induced oxidative stress and neurotoxicity (Koldamova et al., 2001; Paula-Lima et al., 2009). The presence of ApoA-I and its extent of lipidation were also found to significantly affect Aβ clearance in an *in vitro* BBB model (Merino-Zamorano et al., 2016; Dal Magro et al., 2019). Study also has shown that overexpression of human ApoA-I in the circulation prevents learning and memory deficits in APP/PS1 mice (Lewis et al., 2010). When ApoA-I was administered exogenously to mice, it was found that their brain Aβ levels were reduced, which alleviated neuroinflammatory responses and prevented learning and memory deficits in mice (Fernández-de Retana et al., 2017; Button et al., 2019). This suggests that ApoA-I is closely related to AD, and exogenous administration of ApoA-I is an important means of AD treatment (Table 5). Therefore, to explore the possible link between circulating ApoA-I levels and AD susceptibility, we conducted this meta-analysis.

**Table 5:** The related mechanism of apolipoprotein A-I involved in AD pathogenesis.

**References**	**Year**	***In vivo* model**	***In vitro* model**	**Mechanism**	**Intervention effect**
Koldamova et al. (2001)	2001		ApoA-I and Aβ Complex	ApoA-I can decrease Aβ aggregation	ApoA-I attenuated Aβ-induced cellular toxicity
Paula-Lima et al. (2009)	2009		Primary Sprague-Dawley rat neuronal cells	Human apolipoprotein A-I binds amyloid-beta and prevents Abeta-induced neurotoxicity	Binding of apoA-I to Aβ affects the morphology of amyloid aggregates and protects neurons from Aβ-induced oxidative stress and neurotoxicity
Lewis et al. (2010)	2010	APP/PS1/AI triple transgenic mice		Partly by attenuating neuroinflammation and cerebral amyloid angiopathy	Overexpression of human apoA-I in the circulation prevents learning and memory deficits in APP/PS1 mice
Merino-Zamorano et al. (2016)	2016		BBB model constructed from primary cerebral endothelial cells	The presence and localization of ApoA1, influenced Aβ clearance in an *in vitro* BBB model	Peripheral ApoA1 reduce the vascular Aβload and the inflammation associated with its deposition
Fernández-de Retana et al. (2017)	2017	APP23-transgenic mouse model		Reduction of Aβ cerebral deposition induced by the peripheral chronic treatment	Reduced cerebral Aβ levels in mice that received ApoA-I, which were accompanied by a lower expression of astrocyte and microglia neuroinflammatory markers
Button et al. (2019)	2019	APP/PS1 mice		ApoA-I deficiency increases cortical amyloid deposition, cerebral amyloid angiopathy, cortical and hippocampal astrogliosis, and amyloid-associated astrocyte reactivity in APP/PS1 mice	ApoA-I-containing HDL can reduce amyloid pathology and astrocyte reactivity to parenchymal and vascular amyloid in APP/PS1 mice
Dal Magro et al. (2019)	2019		Using immortalized human brain endothelial cells (hCMEC/D3 cells)	The Extent of Human Apolipoprotein A-I Lipidation Strongly Affects the β-Amyloid Efflux Across the Blood-Brain Barrier *in vitro*	When ApoA-I folded its structure in discoidal HDL, rather than in spherical ones, it was able to cross the BBB *in vitro* and strongly destabilize the conformation of Aβ fibrils by decreasing the order of the fibril structure (−24%) and the β-sheet content (−14%)

*ApoA-I, apolipoprotein A-I; Aβ, amyloid β; APP, amyloid precursor protein; PS1, presenilin 1; BBB, blood-brain barrier; HDL, high-density lipoprotein*.

In our study, taking into account the ApoA-I levels in serum, plasma and cerebrospinal fluid, we found that ApoA-I levels were closely related to AD on the basis of the overall circulating levels. The results of our analysis showed a significant association between reduced serum and plasma ApoA-I levels and AD risk. Standardized testing methods may be more helpful in obtaining accurate results and more meaningful indications of conclusions. There was no significant correlation between ApoA-I levels in CSF and AD risk, but ApoA-I levels in cerebrospinal fluid of AD patients showed an upward trend. On the one hand, it may be related to the decline of BBB function in AD state, and on the other hand, it may be related to the clearance of Aβ in the brain. This needs to include more studies to analyze and validate.

The meta-analysis we conducted still has certain limitations. For example, a high degree of heterogeneity was observed in the included studies, possibly due to differences in sample size, measurement methods, and the ethnic background of participants. Four studies did not specify specific measurement methods. Therefore, we performed a subgroup analysis based on the ApoA-I detection method. The results showed that in the subgroup analysis of detection methods in peripheral blood, the NR group had little effect on the overall heterogeneity. In the subgroup analysis of plasma detection methods, there were a total of five studies, and the NR group included three, so it was the main source of heterogeneity, and more studies should be included in the analysis again. There was only one study on CDR scores, one study on low/high Aβ, and two studies on ApoE3/3 and ApoE4/3 genotyping. Due to insufficient data, we could not perform subgroup analyses to study the effect of a specific stratum on heterogeneity and overall outcomes. The number of AD patients in most studies was inconsistent with the number of controls, and individual studies did not reflect the age and sex ratio of the subjects, which may have a certain impact on our results. In addition, none of the included studies reported gender-specific ApoA-I levels, which prevented us from exploring the role of gender in regulating ApoA-I levels.

## Conclusions

In summary, our analysis concluded that serum and plasma levels of ApoA-I were significantly lower in AD patients than in HCs, providing more evidence that ApoA-I has the potential to be a circulating biomarker associated with AD. And ApoA-I supplementation may play a promising preventive or therapeutic strategy for the treatment of AD patients with low ApoA-I levels.

## Data Availability Statement

The datasets presented in this study can be found in online repositories. The names of the repository/repositories and accession number(s) can be found in the article/Supplementary Material.

## Author Contributions

J-hT and M-yL contributed to the conception and design of the study. J-hT and S-qG searched the databases, analyzed the data, and drafted the manuscript. J-hT, S-qG, Y-sZ, and J-rD screened the publications, conducted the quality assessment of the included studies, and extracted the data. M-yL had primary responsibility for the final content. All authors contributed to the writing, reviewing, revising of the manuscript, and read and approved the final manuscript.

## Funding

This work was supported by the National Natural Science Foundation of China (No. 81901309), Key R&D Plan Guidance Project of Liaoning Province (No. 2018225089), Doctoral Research Startup Fund Project of Liaoning Province (No. 2019-BS-289), Key R&D Project of Liaoning Province (No. ZF2019037); 2021 Fundamental Scientific Research Project of Liaoning Province Colleges and Universities (Key Project + General Project) Molecular mechanism of Firmicutes product TMAO driving mitochondrial dynamics to regulate microglial M1/M2 phenotype transformation and affecting AD neuron microenvironment (LJKZ0775), project leader, 2022.01-2024.12, 150,000 yuan; 2021 Double First-Class landmark achievement support plan (123-3110210138), person in charge, 2021.03-2022.03 (50,000 yuan).

## Conflict of Interest

The authors declare that the research was conducted in the absence of any commercial or financial relationships that could be construed as a potential conflict of interest.

## Publisher's Note

All claims expressed in this article are solely those of the authors and do not necessarily represent those of their affiliated organizations, or those of the publisher, the editors and the reviewers. Any product that may be evaluated in this article, or claim that may be made by its manufacturer, is not guaranteed or endorsed by the publisher.

## Abbreviations

AD: Alzheimer's disease
ApoA-I: Apolipoprotein A-I
A: Asian
C: Caucasian
ELISA: enzyme-linked immunosorbent assay
MALDI-TOF MS: Matrix-Assisted Laser Desorption/Ionization Time-of-Flight Mass Spectrometry
NINCDS–ADRDA: National Institute of Neurological and Communicative Disorders and Stroke–Alzheimer's Disease and Related Disorders Association
DSM III: Diagnostic and Statistical Manual of Mental Disorders 3rd Edition
DSM-IV: Diagnostic and Statistical Manual of Mental Disorders 4th Edition
CSF: Cerebrospinal fluid
CDR: Clinical Dementia Rating
MMSE: Mini Mental State Examination
LOAD: Late onset AD
EOAD: Early onset AD
NS: not significant
NR: Not reported
CI: confidence interval
HCs: healthy controls
SMD: standardized mean difference.
