# Supplementary material for: Association of Circulating Apolipoprotein AI Levels in Patients With Alzheimer's Disease: A Systematic Review and Meta-Analysis
Source: Front Aging Neurosci. 2022 May 18;14:899175. doi: 10.3389/fnagi.2022.899175 (PMC9157647; doi:10.3389/fnagi.2022.899175)
Supplement: Supplementary file 2 [file Data_Sheet_2.docx]

**Supplementary Material**

**Contents**

**Supplemental Methods:** Search strategy

**Supplemental Table 1** Newcastle Ottawa Scale scores of the included studies

**Supplemental Table 2** Sensitivity analyses for ApoA-I levels in serum comparing AD Versus HC: excluding one study at a time

**Supplemental Table 3** Publication bias ApoA-I levels in serum comparing AD Versus HC.

**Supplemental Table 4** Sensitivity analyses for ApoA-I levels in plasma comparing AD Versus HC: excluding one study at a time

**Supplemental Table 5** Publication bias ApoA-I levels in plasma comparing AD Versus HC.

**Supplemental Table 6** Sensitivity analyses for ApoA-I levels in peripheral blood comparing AD Versus HC: excluding one study at a time

**Supplemental Table 7** Publication bias for ApoA-I levels in peripheral blood comparing AD Versus HC.

**Supplemental Reference**

**Supplemental Methods**

**Search strategy**

| **No.** | **Search Terms** |
| --- | --- |
| #1 | “Alzheimer’s disease (Title/Abstract) |
| #2 | “ Apolipoprotein AI” (Title/Abstract) |
| #3 | “serum” (Title/Abstract) OR “plasma” (Title/Abstract) OR “ cerebrospinal fluid” (Title/Abstract) |
| #4 | Combination 1 AND 2 AND 3 |

**Supplemental Table 1** Newcastle Ottawa Scale scores of the included studies

| **ID** | **Author** | **Year** | **Population** | **Selection** | | | | **Comparability** | | **Exposure** | | | **Overall  Quality** |
| --- | --- | --- | --- | --- | --- | --- | --- | --- | --- | --- | --- | --- | --- |
|  |  |  |  | 1 | 2 | 3 | 4 | 5A | 5B | 6 | 7 | 8 |  |
| 1 | Kuriyama M[1] | 1992 | Japan | * | * | * | * | * |  | * | * |  | 7 |
| 2 | Kuriyama M[2] | 1994 | Japan | * | * | * | * | * | * | * | * |  | 8 |
| 3 | Merched A[3] | 2000 | France | * | * | * | * | * | * | * | * |  | 8 |
| 4 | Yamamoto H[4] | 2005 | Japan | * | * | * | * | * | * | * | * |  | 8 |
| 5 | Liu HC[5] | 2006 | China | * |  | * | * | * | * | * | * |  | 7 |
| 6 | Xiao Z[6] | 2012 | China | * | * | * | * | * | * | * | * |  | 8 |
| 7 | Lin Q[7] | 2015 | China | * | * | * | * |  |  | * | * |  | 6 |
| 8 | Choi HJ[8] | 2016 | Korea | * |  | * | * | * | * | * | * |  | 7 |
| 9 | Ya L[9] | 2017 | China | * | * | * | * | * | * | * | * |  | 8 |
| 10 | Kawano M[10] | 1995 | Japan | * |  | * | * | * | * | * | * |  | 7 |
| 11 | Bergt C[11] | 2006 | USA | * | * | * | * | * | * | * | * |  | 8 |
| 12 | Khalil A[12] | 2012 | Canada | * | * | * | * | * | * | * | * |  | 8 |
| 13 | Yang SY[13] | 2015 | China | * | * | * | * | * | * | * | * |  | 8 |
| 14 | Slot RE[14] | 2017 | Netherland | * | * | * | * | * | * | * | * |  | 8 |
| 15 | Song H[15] | 1997 | Japan | * | * | * |  | * | * | * | * |  | 7 |
| 16 | Kindy MS[16] | 1999 | USA | * |  | * | * |  |  | * | * |  | 5 |
| 17 | Demeester N[17] | 2000 | Belgium | * | * | * | * |  |  | * | * |  | 6 |
| 18 | Yassine HN[18] | 2016 | USA | * |  | * | * | * | * | * | * |  | 7 |

1. Case definition is sufficient, with independent verification. 2. Continuous collection and representative cases. 3. Community control. 4. Control of disease history without neurological system. 5 A. Research controls age. 5 B. Research controls other confounding factors. 6. Exposure is determined by reliable records. 7. The same method was used to determine the exposure of the case group and the control group. 8. None-response rates were similar for case and control groups. A study can be awarded a maximum of one star for each numbered item within the Selection and Exposure categories and a maximum of two stars for Comparability. Scores for low (0–3), moderate (4–6), and high-quality studies (7–9) were assigned.

**Supplemental Table 2** Sensitivity analyses for ApoA-I levels in serum comparing AD Versus HC: excluding one study at a time^1^

| **Study omitted** | **Estimate** | **[95% Conf. Interval]** | |
| --- | --- | --- | --- |
| Kuriyama M et al. (1992) [1] | -1.274 | -1.846 | -0.701 |
| Kuriyama M et al. (1994) [2] | -1.189 | -1.797 | -0.582 |
| Merched A et al. (2000) [3] | -1.162 | -1.786 | -0.537 |
| Merched A et al.(ApoE3/3) (2000) | -1.150 | -1.757 | -0.543 |
| Merched A et al.(ApoE4/3) (2000) | -1.179 | -1.775 | -0.582 |
| Yamamoto H et al. (2005) [4] | -1.222 | -1.817 | -0.628 |
| Liu HC et al. (2006) [5] | -1.122 | -1.736 | -0.508 |
| Xiao Z et al. (2012) [6] | -1.236 | -1.809 | -0.663 |
| Lin Q et al.(CDR 0.5) (2015) [7] | -1.100 | -1.715 | -0.486 |
| Lin Q et al.(CDR 1) (2015) | -1.065 | -1.651 | -0.480 |
| Lin Q et al.(CDR 2) (2015) | -1.030 | -1.596 | -0.465 |
| Choi HJ et al.(low Aβ) (2016) [8] | -1.215 | -1.807 | -0.623 |
| Choi HJ et al.(high Aβ) (2016) | -1.247 | -1.831 | -0.664 |
| Ya L et al. (2017) [9] | -0.983 | -1.481 | -0.485 |
| Combined | -1.155 | -1.722 | -0.588 |

^1^Effect sizes were pooled using random-effects mode

**Supplemental Table 3** Publication bias ApoA-I levels in serum comparing AD Versus HC.

**A**

| Begg's Test | | |
| --- | --- | --- |
| adj. Kendall's Score (P-Q) | = | 7.000 |
| Std. Dev. of Score | = | 18.270 |
| Number of Studies | = | 14.000 |
| z | = | 0.380 |
| Pr > \|z\| | = | 0.702 |
| z | = | 0.33 (continuity corrected) |
| Pr > \|z\| | = | **0.743** (continuity corrected) |

**B**

| Egger's test | | | | | | |
| --- | --- | --- | --- | --- | --- | --- |
| Std_Eff | Coef. | Std.Err | t | P>\|t\| | [95% Conf.Interval] | |
| slope | -1.262 | 1.138 | -1.110 | 0.289 | -3.742 | 1.218 |
| bias | 0.305 | 5.299 | 0.060 | **0.955** | -11.240 | 11.849 |

**Supplemental Table 4** Sensitivity analyses for ApoA-I levels in plasma comparing AD Versus HC: excluding one study at a time^1^

| **Study omitted** | **Estimate** | **[95% Conf. Interval]** | |
| --- | --- | --- | --- |
| Kawano M et al.(ApoE3/3) (1995) [10] | -1.132 | -2.220 | -0.043 |
| Kawano M et al.(ApoE4/3) (1995) | -1.174 | -2.236 | -0.111 |
| Bergt C et al. (2006) [11] | -1.358 | -2.475 | -0.241 |
| Khalil A et al. (2012) [12] | -0.514 | -1.016 | -0.011 |
| Yang SY et al. (2015) [13] | -1.261 | -2.406 | -0.116 |
| Slot RE et al. (2017) [14] | -1.391 | -2.541 | -0.241 |
| Combined | -1.130 | -2.046 | -0.213 |

^1^Effect sizes were pooled using random-effects mode

**Supplemental Table 5** Publication bias ApoA-I levels in plasma comparing AD Versus HC.

**A**

| Begg's Test | | |
| --- | --- | --- |
| adj. Kendall's Score (P-Q) | = | -9.000 |
| Std. Dev. of Score | = | 5.320 |
| Number of Studies | = | 6.000 |
| z | = | -1.690 |
| Pr > \|z\| | = | 0.091 |
| z | = | 1.50(continuity corrected) |
| Pr > \|z\| | = | **0.133**(continuity corrected) |

**B**

| Egger's test | | | | | | |
| --- | --- | --- | --- | --- | --- | --- |
| Std_Eff | Coef. | Std.Err | t | P>\|t\| | [95% Conf.Interval] | |
| slope | 0.703 | 0.515 | 1.360 | 0.244 | -0.727 | 2.132 |
| bias | -5.717 | 2.390 | -2.390 | **0.075** | -12.352 | 0.918 |

**Supplemental Table 6** Sensitivity analyses for ApoA-I levels in peripheral blood comparing AD Versus HC: excluding one study at a time^1^

| **Study omitted** | **Estimate** | **[95% Conf. Interval]** | |
| --- | --- | --- | --- |
| Kuriyama M et al. (1992) [1] | -1.229 | -1.722 | -0.736 |
| Kuriyama M et al. (1994) [2] | -1.172 | -1.682 | -0.662 |
| Merched A et al. (2000) [3] | -1.153 | -1.671 | -0.636 |
| Merched A et al.(ApoE3/3) (2000) | -1.145 | -1.651 | -0.638 |
| Merched A et al.(ApoE4/3) (2000) | -1.164 | -1.666 | -0.662 |
| Yamamoto H et al. (2005) [4] | -1.194 | -1.700 | -0.689 |
| Liu HC et al. (2006) [5] | -1.125 | -1.633 | -0.618 |
| Xiao Z et al. (2012) [6] | -1.203 | -1.712 | -0.693 |
| Lin Q et al.(CDR 0.5) (2015) [7] | -1.110 | -1.614 | -0.607 |
| Lin Q et al.(CDR 1) (2015) | -1.085 | -1.573 | -0.596 |
| Lin Q et al.(CDR 2) (2015) | -1.059 | -1.536 | -0.582 |
| Choi HJ et al.(low Aβ) (2016) [8] | -1.189 | -1.690 | -0.688 |
| Choi HJ et al.(high Aβ) (2016) | -1.211 | -1.709 | -0.714 |
| Ya L et al. (2017) [9] | -1.021 | -1.456 | -0.586 |
| Kawano M et al.(ApoE3/3) (1995) [10] | -1.147 | -1.651 | -0.643 |
| Kawano M et al.(ApoE4/3) (1995) | -1.157 | -1.658 | -0.656 |
| Bergt C et al. (2006) [11] | -1.203 | -1.702 | -0.704 |
| Khalil A et al. (2012) [12] | -0.998 | -1.470 | -0.527 |
| Yang SY et al. (2015) [13] | -1.178 | -1.682 | -0.674 |
| Slot RE et al. (2017) [14] | -1.211 | -1.702 | -0.720 |
| Combined | -1.148 | -1.631 | -0.664 |

^1^Effect sizes were pooled using random-effects mode

**Supplemental Table 7** Publication bias for ApoA-I levels in peripheral blood comparing AD Versus HC.

**A**

| Begg's Test | | |
| --- | --- | --- |
| adj. Kendall's Score (P-Q) | = | -8 |
| Std.Dev.of Score | = | 30.82 |
| Number of Studies | = | 20 |
| z | = | -0.26 |
| Pr > \|z\| | = | 0.795 |
| z | = | 0.23 (continuity corrected) |
| Pr > \|z\| | = | 0.820 (continuity corrected) |

**B**

| Egger's test | | | | | | |
| --- | --- | --- | --- | --- | --- | --- |
| Std_Eff | Coef. | Std.Err | t | P>\|t\| | [95% Conf.Interval] | |
| slope | -0.108 | 0.654 | -0.17 | 0.870 | -1.483 | 1.266 |
| bias | -4.176 | 3.047 | -1.37 | 0.187 | -10.578 | 2.226 |

**Supplemental Reference**

[1] Kuriyama M, Hokezu Y, Togo S *et al.* [Serum lipids, lipoproteins and apolipoproteins in patients with senile dementia]. Nihon Ronen Igakkai zasshi. Japanese journal of geriatrics 1992; 29:559-564.

[2] Kuriyama M, Takahashi K, Yamano T *et al.* Low levels of serum apolipoprotein A I and A II in senile dementia. The Japanese journal of psychiatry and neurology 1994; 48:589-593.

[3] Merched A, Xia Y, Visvikis S *et al.* Decreased high-density lipoprotein cholesterol and serum apolipoprotein AI concentrations are highly correlated with the severity of Alzheimer's disease. Neurobiology of aging 2000; 21:27-30.

[4] Yamamoto H, Watanabe T, Miyazaki A *et al.* High prevalence of Chlamydia pneumoniae antibodies and increased high-sensitive C-reactive protein in patients with vascular dementia. Journal of the American Geriatrics Society 2005; 53:583-589.

[5] Liu HC, Hu CJ, Chang JG *et al.* Proteomic identification of lower apolipoprotein A-I in Alzheimer's disease. Dementia and geriatric cognitive disorders 2006; 21:155-161.

[6] Xiao Z, Wang J, Chen W *et al.* Association studies of several cholesterol-related genes (ABCA1, CETP and LIPC) with serum lipids and risk of Alzheimer's disease. Lipids in health and disease 2012; 11:163.

[7] Lin Q, Cao Y, Gao J. Decreased expression of the APOA1-APOC3-APOA4 gene cluster is associated with risk of Alzheimer's disease. Drug design, development and therapy 2015; 9:5421-5431.

[8] Choi HJ, Seo EH, Yi D *et al.* Amyloid-Independent Amnestic Mild Cognitive Impairment and Serum Apolipoprotein A1 Levels. The American journal of geriatric psychiatry : official journal of the American Association for Geriatric Psychiatry 2016; 24:144-153.

[9] Ya L, Lu Z. Differences in ABCA1 R219K Polymorphisms and Serum Indexes in Alzheimer and Parkinson Diseases in Northern China. Medical science monitor : international medical journal of experimental and clinical research 2017; 23:4591-4600.

[10] Kawano M, Kawakami M, Otsuka M *et al.* Marked decrease of plasma apolipoprotein AI and AII in Japanese patients with late-onset non-familial Alzheimer's disease. Clinica chimica acta; international journal of clinical chemistry 1995; 239:209-211.

[11] Bergt C, Nakano T, Ditterich J *et al.* Oxidized plasma high-density lipoprotein is decreased in Alzheimer's disease. Free radical biology & medicine 2006; 41:1542-1547.

[12] Khalil A, Berrougui H, Pawelec G, Fulop T. Impairment of the ABCA1 and SR-BI-mediated cholesterol efflux pathways and HDL anti-inflammatory activity in Alzheimer's disease. Mechanisms of ageing and development 2012; 133:20-29.

[13] Yang SY, Shan CL, Qing H *et al.* The Effects of Aerobic Exercise on Cognitive Function of Alzheimer's Disease Patients. CNS & neurological disorders drug targets 2015; 14:1292-1297.

[14] Slot RE, Van Harten AC, Kester MI *et al.* Apolipoprotein A1 in Cerebrospinal Fluid and Plasma and Progression to Alzheimer's Disease in Non-Demented Elderly. Journal of Alzheimer's disease : JAD 2017; 56:687-697.

[15] Song H, Saito K, Seishima M *et al.* Cerebrospinal fluid apo E and apo A-I concentrations in early- and late-onset Alzheimer's disease. Neuroscience letters 1997; 231:175-178.

[16] Kindy MS, Yu J, Guo JT, Zhu H. Apolipoprotein Serum Amyloid A in Alzheimer's Disease. Journal of Alzheimer's disease : JAD 1999; 1:155-167.

[17] Demeester N, Castro G, Desrumaux C *et al.* Characterization and functional studies of lipoproteins, lipid transfer proteins, and lecithin:cholesterol acyltransferase in CSF of normal individuals and patients with Alzheimer's disease. Journal of lipid research 2000; 41:963-974.

[18] Yassine HN, Feng Q, Chiang J *et al.* ABCA1-Mediated Cholesterol Efflux Capacity to Cerebrospinal Fluid Is Reduced in Patients With Mild Cognitive Impairment and Alzheimer's Disease. Journal of the American Heart Association 2016; 5.
